# Predictability of Precipitation Over the Conterminous U.S. Based on the CMIP5 Multi-Model Ensemble

**DOI:** 10.1038/srep29962

**Published:** 2016-07-18

**Authors:** Mingkai Jiang, Benjamin S. Felzer, Dork Sahagian

**Affiliations:** 1 Earth and Environmental Sciences, Lehigh University, 1 W Packer Avenue, Bethlehem, PA, 18015, United States

## Abstract

Characterizing precipitation seasonality and variability in the face of future uncertainty is important for a well-informed climate change adaptation strategy. Using the Colwell index of predictability and monthly normalized precipitation data from the Coupled Model Intercomparison Project Phase 5 (CMIP5) multi-model ensembles, this study identifies spatial hotspots of changes in precipitation predictability in the United States under various climate scenarios. Over the historic period (1950–2005), the recurrent pattern of precipitation is highly predictable in the East and along the coastal Northwest, and is less so in the arid Southwest. Comparing the future (2040–2095) to the historic period, larger changes in precipitation predictability are observed under Representative Concentration Pathways (RCP) 8.5 than those under RCP 4.5. Finally, there are region-specific hotspots of future changes in precipitation predictability, and these hotspots often coincide with regions of little projected change in total precipitation, with exceptions along the wetter East and parts of the drier central West. Therefore, decision-makers are advised to not rely on future total precipitation as an indicator of water resources. Changes in precipitation predictability and the subsequent changes on seasonality and variability are equally, if not more, important factors to be included in future regional environmental assessment.

Changes in precipitation associated with future climate change will strongly impact ecological processes and the utilization of ecosystem services. As projected by the Phase 5 of the Coupled Model Intercomparison Project (CMIP5) of the Intergovernmental Panel on Climate Change (IPCC) and the United States (US) National Climate Assessment^1^,^2^, precipitation in the US is expected to change in a spatially and temporally heterogeneous manner in the 21^st^ century^3^,^4^,^5^,^6^,^7^,^8^,^9^,^10^. We are currently observing rapid changes in Earth’s hydrological cycle, and plans for optimizing societal infrastructural and environmental resilience in the face of future changes depend on the proper descriptions of future precipitation fluctuation and seasonality at the regional scale in this rapidly changing world^1^. Changes in precipitation fluctuation and seasonality will affect resource availability, which will ultimately impact biodiversity and the decisions to manage hydrological, agricultural, and ecological systems. Current efforts assessing climate change-induced precipitation redistribution focus on long-term temporal trends and seasonal patterns. Such predictions are important for well-informed adaptation and mitigation decision-making, yet there is also a need to investigate whether the perturbed precipitation patterns will become more or less predictable (in terms of how seasonal precipitation pattern fluctuate inter-annually) for natural adaptation to take place. This has led to a knowledge gap in terms of an ecologically and/or hydrologically meaningful characterization of changes of precipitation variability. This study is directed toward providing an initial step toward rectifying this knowledge gap.

A variable climate is not necessarily an unpredictable one, as variability consists of both stochasticity and cyclicity^11^. Maximum stochasticity results from perfectly random fluctuation, while a cyclic fluctuation is represented by a time-dependent occurrence probability. As such, the combination of stochasticity and cyclicity defines the likelihood of an expected climatic condition, which in turn defines how predictable a particular pattern is, and the consequent environmental stability/regularity that drives ecosystem structure and functionality. Environmental cues are certain predictable factors (e.g. photoperiod, temperature, precipitation) providing reliable signals to anticipate optimal agricultural options^12^,^13^, breeding conditions^14^, succession^15^, wildlife adaptation strategies^16^ as well as human system infrastructure^17^. In a perturbed climate, such as those simulated under various RCP greenhouse gas scenarios, precipitation stochasticity and cyclicity will likely change, resulting in altered precipitation predictability from present. Proper understanding of the changes in the precipitation predictability expected to result from global change is fundamental to the sustainable utilization of ecosystem services, and presents one of the greatest challenges to humanity in the face of future uncertainty^18^. The goal of the current study has therefore been to characterize the changes in precipitation predictability, so as to gain insights regarding changes in the likelihood of recurrent precipitation patterns that control ecosystem dynamics and the provision of ecosystem services.

The Colwell index^19^, also referred to as the constancy/contingency model^14^, utilizes information theory to provide measures of the likelihood of an expected phenomenon to occur, so as to reveal the properties of predictability nested within the long-term data records (e.g. climate^20^; species life-history trait evolution^16^; hydrological flow variation)^21^. The Colwell index of predictability (P) can be decomposed into two terms: constancy (C) and seasonality (S). An environmental phenomenon can be predictable because it has constancy over time (i.e. there is little to no magnitude change over any timescale), or because the degree to which the quantity changes depends upon season, but is consistent inter-annually (i.e. seasonality)^14^. The Colwell index thus provides simple yet interpretable quantification of stochasticity and cyclicity to improve understanding of precipitation predictability based on observed seasonal and inter-annual variability from existing climate data^22^,^23^. Consequently, the Colwell index is adopted in this study to explore properties of observed (i.e. Maurer historic dataset)^24^,^25^ and CMIP5-simulated precipitation predictability. Specifically, this study seeks to: 1) provide spatial patterns of precipitation predictability over the conterminous US landscape; 2) evaluate the performance of the CMIP5 multi-model ensemble in simulating precipitation predictability over the period 1950–2005; and 3) identify spatial hotspots of changes in precipitation predictability in the US under various future climate change scenarios (i.e. RCP 4.5 and RCP 8.5). This study generalizes and builds on Jiang *et al*.^23^, which only investigated spatial patterns of precipitation extremes over the historic period. The term “predictability” used in this study differs from traditional understanding of predictability in that it does not reflect the predictive power of modeled precipitation based on understanding of the underlying processes and mechanisms, but rather, it is a description of the power of past precipitation attributes in both seasonality (contingency) and inter-annual variability within a specific time interval (constancy throughout 1950–2005) of observed precipitation itself to predict future precipitation.

## Results

### Spatial patterns of precipitation predictability

**Figure 1:**
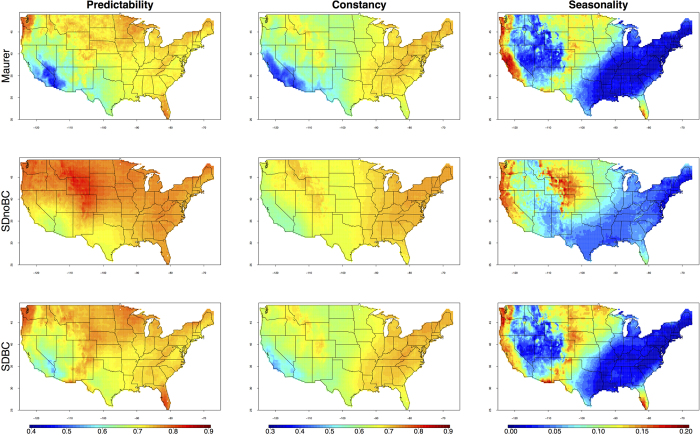
Gridded monthly precipitation predictability, constancy, and seasonality over the conterminous US for the period 1950–2005, based on Maurer, CMIP5 Spatially Downscaled but not Bias Corrected (SDnoBC), and CMIP5 Spatially Downscaled and Bias corrected (SDBC) monthly precipitation datasets. Figure was plotted in R Studio (V0.99, RStudio Inc.).

Clear regional contrasts for the distributions of precipitation P, C and S are observed (Fig. 1, top row). In general, over the period 1950–2005, precipitation is less predictable in the arid Southwest as compared to the East and the coastal Pacific Northwest. Further, a decreasing pattern from the Southwest to the Northeast is apparent for scores of C (high scores indicate high magnitude constancy). Additionally, parts of the Interior West along the Rocky Mountains also have relatively invariant precipitation fluctuations (little change in interannual variability). In comparison, the spatial distributions of S have much sharper regional contrasts: relatively higher scores of S are observed in Florida, parts of the Midwest, and along the West coast.

### Capacity of Earth system models

**Figure 2:**
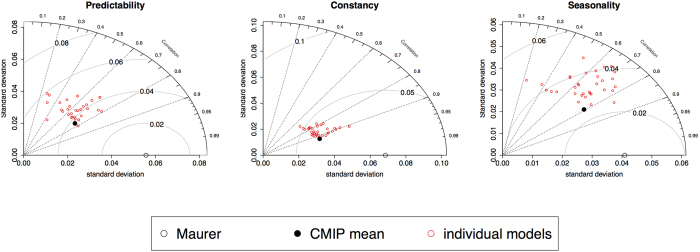
Taylor plot for the computed predictability, constancy, and seasonality scores across the US landscape for the historic period 1950–2005, based on Maurer monthly precipitation (open black circle), CMIP5 Spatial Downscaled but not Bias Corrected (SDnoBC) multi-model ensemble means (closed black circle), and the individual CMIP5 SDnoBC model results (open red circles). Figure was plotted in R Studio (V0.99, RStudio Inc.).

For the same timeframe (i.e. 1950–2005), the Colwell index based on CMIP5 SDnoBC data show a wide range of correlations to those computed from historic observations (correlation coefficients between individual CMIP5 models to Maurer dataset range between 0.2–0.8, 0.7–0.9, and 0.2–0.85 for scores of P, C and S, respectively) (Fig. 2). Spatially, historic distributions of P, C and S are not well represented by the SDnoBC multimodel means (Fig. 1, middle row). For instance, there is a reduced Southwest-Northeast contrast for the scores of C in the SDnoBC dataset compared to those generated by the Maurer dataset, and it is also evident that precipitation P in the Intermountain West is generally less well represented by the CMIP5 models. Spatial representations of individual model results based on SDnoBC dataset are provided in Figure S1. Bias-correction substantially improves the correlation between the CMIP5 multimodel ensemble and historic observations (Figure S2), with some minor regional discrepancies (Fig. 1, bottom row). Based on the SDBC dataset, it is thus possible to provide comparisons of precipitation predictability between historic baseline and the future under various RCP scenarios.

### Hotspots of changes in precipitation predictability

**Figure 3:**
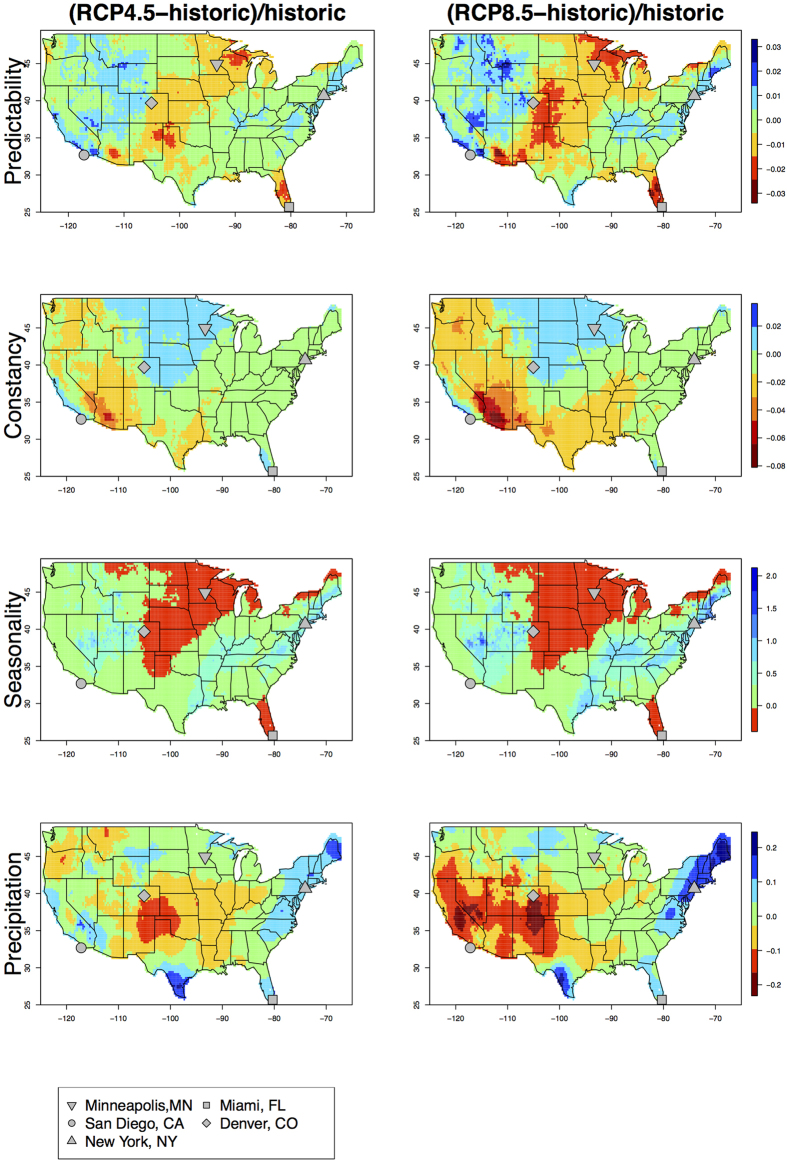
Gridded percent differences for scores of predictability, constancy, seasonality, and mean annual precipitation percent differences over the conterminous US between the future RCP4.5 scenario and historic periods (left panel), and between the future RCP8.5 scenario and historic periods (right panel), based on the CMIP5 multi-model ensemble Spatially Downscaled and Bias Corrected (SDBC) monthly precipitation datasets. Figure was plotted in R Studio (V0.99, RStudio Inc.).

Comparisons of changes in precipitation predictability between the historic baseline and the future under the two RCP scenarios are provided in Fig. 3. More significant changes of P, C and S occur in RCP 8.5 than in the RCP 4.5 scenario (Z-value and P-value for statistical significance of the change are provided in Figures S3 and S4, respectively). Precipitation becomes less predictable (as high as 3% reduction) in Florida and the Central US, and becomes more predictable in sporadic locations of the West (as high as 3% increase). Precipitation fluctuations become less constant in the West (except the southern California coast) and the Central South (as high as 8% decrease in C scores, which represents increased variability), and become more constant in the Central North (2% increase in C scores). In comparison, reductions in S are observed in the upper Midwest, northern Great Plains, and Florida (~ − 50%), and enhanced S are apparent in the desert Southwest and along the path of frequent storm tracks on the East Coast (~ + 200%).

Changes in mean annual precipitation between the historic and future periods show different spatial patterns compared to those changes in P (Fig. 3, bottom row). Under the RCP4.5 scenario, mean annual precipitation in the future (i.e. 2040–2095) increased by 20% in the Northeast and parts of the South (e.g. south corner of Texas and Florida), and decreased by ~20% in the central Great Plains. Under the RCP8.5 scenario, similar patterns are observed across the US, with the exception of the West (especially California), where reductions in mean annual precipitation occur.

## Discussion

A generally decreasing pattern of precipitation predictability from the Northeast to the Southwest is identified in this study across the US landscape (Fig. 1). Despite its relatively low magnitude, precipitation in hot, arid deserts is frequently described in qualitative terms such as “unpredictable” for its high stochasticity between precipitation and non-precipitation events^26^,^27^. This study, using standardized precipitation data (i.e. calculated as monthly percent of annual total to allow meaningful comparison between wet and dry regions), finds that precipitation in the Southwest arid/semi-arid region is indeed less predictable than other parts of the country, largely as a result of low scores of constancy (and therefore highly variable). However, precipitation seasonality in this region exhibits a strong coast-interior contrast: the West coast has some of the highest S scores across the US landscape, whereas the interior West has some of the lowest. As such, precipitation is unpredictable in the interior Southwest for its low magnitude constancy and seasonality, while it is unpredictable in coastal California only because of its low constancy.

Moreover, precipitation is highly predictable in the East and along the coastal Pacific Northwest as a result of invariant precipitation magnitude fluctuation both seasonally and inter-annually. For Florida, a stronger seasonality is observed, reflecting the known wet (summer) and dry (from mid fall through late spring) seasons of its sub-tropical climate^28^. Along the Pacific Northwest coast, precipitation is also highly predictable, with relatively high C and S scores. The observed strong seasonality along the Pacific Northwest coast reflects its typical wet winter and dry summer climate^29^,^30^.

The interpretation of the estimated precipitation predictability at the national level must consider that the calculation of the Colwell index is scale-dependent: the inclusion/exclusion of different geographic range and/or different timeframe will affect the computed scores of predictability^31^. This study uses 56-year of monthly precipitation data at the national level to minimize the impact of phase changes in large-scale climatic oscillations (e.g. Pacific Decadal Oscillation or El Nino Southern Oscillation). Additionally, this study standardizes monthly precipitation as percent of annual total, so that the classification scheme to compute the Colwell index is standardized across all grids in the US. By doing this, inclusion/exclusion of different geographic ranges does not affect the computed Colwell index, and the 56-year of data provides a reliable range of time to define the predictability of a long-term climate that is meaningful for providing stable environmental cues for ecological adaptation and evolution.

Our understanding of future climate change is largely based on projections from state-of-the-art earth system models, but there are still large uncertainties in model simulations^32^. Earth system models are increasingly able to realistically simulate spatial distribution and temporal changes of precipitation means and extremes^33^,^34^,^35^. While it has been demonstrated that models are able to provide robust estimates of the magnitude and directional shift in climate change^35^, limited information is available quantifying the model uncertainties in simulating climate variability. A recent call for including variability in climate change assessment has been made^36^, arguing that including variability in future climate change analyses would allow the differentiation of normal and abnormal events, thereby providing an indication of the changes in predictability. Based on the SDnoBC datasets of the CMIP5 multimodel ensemble, this study shows current Earth system models are not yet able to accurately estimate precipitation variability (the reverse of constancy), seasonality, and predictability (Fig. 2). The discrepancy is especially large in the interior West and the Central US (Fig. 1), reflecting the possible limitations of models to simulate air-land interactions in complex topography.

Climate variability across a range of timescales determines the structure and function of Earth’s ecosystems. Over the last several hundred years in which humans have been making scientific observations of the environment, actual changes in climate and the hydrological cycle have been relatively small. There is, however, unequivocal evidence that Earth’s hydrological cycle is now changing at an unprecedented rate, and our existing societal infrastructure and environmental resilience depends on knowledge of how predictable future precipitation would be in this rapidly changing world^1^. Undoubtedly, the magnitude and predictability of the change are both important to understand for a well-informed climate change adaptation and mitigation strategy in the face of future uncertainty. Below, three examples are given to illustrate the implications of precipitation predictability regarding biodiversity conservation, crop production, and urban systems across the conterminous US landscape.

The California Floristic Province has been identified as an endemic plant hotspot of global importance^37^,^38^. The combination of California’s complex geological history, sharp climatic gradients, and climatic fluctuations generated by changes in ocean currents has allowed genetic diversification in the region over the past several millions of years^39^. Climatic factors, especially precipitation, were the strongest predictors of elevated biodiversity within the Province^40^,^41^. This study shows that the favorably consistent precipitation conditions that allowed the persistence and diversification of endemic species in California is likely to become temporally less constant in the future over the Sierra Nevada mountains. Superimposed on top of this are likely enhanced aridification under the RCP8.5 scenario and reduced aridification under the RCP4.5 scenario, rendering not only the magnitude, but also the sign of predicted changes dependent on emissions scenarios. Although there is little change in total predictability scores, the expected changes in precipitation constancy and totals have two important biodiversity conservation implications: 1) reduced constancy implies more variable and hence possibly more rare climatic events to obscure the interpretation of environmental cues (e.g. resource availability) in choosing strategies for reproduction in mammals and birds (e.g. female length at first reproduction and longevity of tule perch – a fish species confined to drainages in central California – varied directly with environmental predictability)^42^, thereby challenging ecosystem resilience and creating less favorable climatic conditions for natural adaptation to climate change; and 2) climate change adaptation policies targeting different emission scenarios would result in completely different biodiversity management options, as aridification is likely to be ameliorated under RCP4.5, but is likely to be exacerbated under RCP8.5 scenario. New conservation policies should holistically consider these implications and uncertainties.

Iowa, Minnesota and Wisconsin in the Midwest are traditional corn production states in the US. According to statistics from the US Department of Agriculture^43^, corn production in year 2014 from these 3 states alone account for ~30% of the US’s annual total production. Studies have shown that corn yield is mostly influenced by precipitation variation, especially over an 8-week of vital growth period^44^. Future changes in precipitation in these regions thus have important food safety implications at the national level.

Across most of the Corn Belt, precipitation has been increasing in the early growing season but decreasing in the late growing season^45^. This within-season precipitation trend correlates spatially (especially in the 3 states mentioned above) with the most prominent reductions in precipitation predictability, accompanied by enhanced C scores and reduced S scores (Fig. 3). The increases in C indicate that precipitation is expected to become more constant interannually, and the reduced S indicates that precipitation is expected to become less seasonal, with important implications for the summer growing season. With little to no change in mean annual precipitation under both RCP4.5 and RCP8.5 scenarios (Fig. 3, bottom panel), precipitation is essentially more constant both within year and among years. However, the reduction in predictability means that while precipitation may not vary seasonally or interannually, the total annual precipitation is relatively unpredictable, and thus could be drastically different than current. Crop policies based on analyses of precipitation directional or magnitude change are inadequate in addressing the issues revealed by changes in seasonality and predictability. Consequently, future water resource management should recognize the importance of considering climate predictability for better-prepared irrigation infrastructure and farming practices.

Changes in precipitation P, C, S and annual totals in five widely separated major US cities are outlined in Table 1 (Minneapolis, San Diego, New York, Miami, and Denver). Comparing the future (2040–2099) to the historic period (1950–2005), mean annual precipitation will increase (1.4–9.5%) in Minneapolis, San Diego, New York and Miami, and will decrease (−3.2%) in Denver under the RCP4.5 scenario. The same directional change in total precipitation is observed for all cities except San Diego under the RCP8.5 scenario. As indicated by the Colwell index, dry seasons in Minneapolis, San Diego (under only RCP 4.5 scenario) and Miami are getting wetter (reduced seasonality accompanied by increases in annual total), but interannual variability is more predictable only in San Diego, implying that more fluctuations will occur in Minneapolis and Miami, in addition to the reduced seasonality and enhanced precipitation totals. In comparison, wet seasons in Denver and San Diego (under the RCP 8.5 scenario) are getting drier (reduced seasonality accompanied by decreases in annual total), but precipitation is becoming more predictable only in San Diego (under RCP 8.5), indicating a seasonally and inter-annually consistent drier trajectory. For New York City, precipitation is becoming more predictable with enhanced seasonality and reduced constancy. At the same time, precipitation total increases, thus wet seasons get wetter while totals vary greatly from year to year.

Consequently, it is implied that a high flood risk may be possible for New York City (depending on how much of the wet-getting-wetter scenario results in extreme precipitations), and a high likelihood of persistent drought in San Diego under the RCP 8.5 scenario. Uncertainties in changes in precipitation are also high as a result of different emission scenarios for San Diego. Nevertheless, existing infrastructure designed to cope with drought and flooding events has been challenged by recent occurrences (e.g. California drought^46^; New York flooding)^17^,^47^, and specific approaches to enhancing infrastructural resilience in these likely future scenarios is critical to local residents, stakeholders and decision makers.

## Conclusion

The Colwell index provides an appropriate tool to complement the traditional methods in revealing climate predictability. Analysis of the predictability of CMIP5 ensemble results with regard to precipitation indicates that precipitation is more predictable in the East and along the Pacific Northwest coast, and is generally less so in the arid Southwest. Furthermore, it is evident that Earth system models without bias-correction are unable to provide retrodictions that match the observed patterns of precipitation predictability, constancy and seasonality, but that bias corrections allow a more realistic comparison between historic and future periods. Comparing the future (2040–2095) to the historic (1950–2005) periods, more prominent changes in precipitation predictability are observed under the RCP 8.5 scenario than those under the RCP 4.5 scenario. Finally, region-specific hotspots of future changes in precipitation predictability are found in California, the Pacific Northwest, and the Great Plains, and these regional hotspots are ecologically and socio-economically important. These changes in predictability do not coincide with changes in projected annual precipitation totals; as such, decision-makers are admonished to not rely on future total precipitation as an indicator of water resources and availability. Changes in precipitation predictability and the subsequent changes on seasonality and interannual variability are equally, if not more, important factors to be included in future regional environmental assessment.

## Materials and Methods

### Datasets

Historic observed monthly precipitation data were based on daily gridded meteorological data for the conterminous US at 1/8° resolution over the period 1950–2005 24,25. Monthly precipitation data of the CMIP5 Spatially Downscaled and Bias Corrected (SDBC) precipitation dataset at 1/8° resolution for the periods 1950–2005 and 2040–2095, and the CMIP5 Spatially Downscaled but not Bias Corrected (SDnoBC) dataset for the period 1950–2005, were utilized^48^,^49^. The bias correction follows a quantile mapping technique operated on a monthly and location-specific basis, which involves using observed and modeled monthly data from 1950–1999 to determine the biases which are then applied to the future climate^49^. The historic period provided a baseline for comparison of the computed Colwell index between historic and future time periods. For the future period the RCP 8.5 and RCP 4.5 scenarios were used to establish a range of likely future trajectories. Models included in this study are provided in Table S1. The monthly precipitation data were transformed into monthly percent of annual total to allow a meaningful comparison between regions of different precipitation totals and interannual variability. The Colwell index was subsequently generated based on the 56-year monthly precipitation datasets at the gridded-level.

### The Colwell Index

The term “predictability” used in this study, as introduced earlier, differs from traditional understanding of predictability in that it is not about the predictive power of precipitation based on understandings of the underlying processes and mechanisms, but more a description of the precipitation variability over a certain timeframe itself. Precipitation predictability^19^ is numerically represented by the sum of magnitude constancy and seasonal contingency (which we call seasonality hereinafter) that varies from 0 (completely unpredictable) to 1 (completely predictable). Constancy is essentially a measure of the precipitation variability that varies inversely with magnitude of precipitation fluctuation, and seasonality is a measure of the seasonal dependence of precipitation through time. A high score of constancy indicates that total annual precipitation will not vary through time at any given locality (regardless of the value of precipitation at that locality), while a high score in seasonality indicates that precipitation is highly seasonal. Scores of predictability (P), constancy (C) and seasonality (S) all ranges between 0–1. Details of the mathematical derivation are provided in SI Text 1.

To apply the Colwell index with monthly precipitation data, a 12 × 12 frequency matrix consisting of 56-year monthly precipitation data was constructed at the gridded level. Each column represents a month within the year, and each row represents a certain level of precipitation. Because the maximum monthly precipitation as percent of annual total is 1, the 12 precipitation bins are classified as 0, 0–1/11, 1/11–2/11, … 10/11–11/11. The 56-year of monthly precipitation data were projected onto the frequency matrix at each grid, using the respective historic and future datasets. Individual CMIP5 model results were aggregated at each grid level to compute the multimodel ensemble mean; hence each model has an equal weight in the final multimodel ensemble mean. Data processing and analyses were performed in R Studio (V0.99, RStudio Inc.). Code scripts are available in SI Text 2.

## Additional Information

**How to cite this article**: Jiang, M. *et al*. Predictability of precipitation Over the conterminous U.S. Based on the CMIP5 Multi-Model Ensemble. *Sci. Rep.* **6**, 29962; doi: 10.1038/srep29962 (2016).

## Supplementary Material

Supplementary Information

Supplementary Information 2a

Supplementary information 2b

## Figures and Tables

**Table 1:** City-specific percent differences for scores of predictability (P), constancy (C), seasonality (S), and mean annual precipitation over the conterminous US between the future RCP 4.5 scenario and historic periods, and between the future RCP 8.5 and historic periods, based on the CMIP5 multi-model Spatially Downscaled and Bias Corrected (SDBC) monthly precipitation datasets.

Case	City	Latitude	Longitude	% diff RCP4.5 - hist				% diff RCP8.5 - hist			
				Annual mean prcp	P	C	S	Annual mean prcp	P	C	S
1	Minneapolis, MN	44.9375	−93.3125	1.4	−1.3	0.8	−12.8	0.4	−1.7	1.3	−19.1
2	San Diego, CA	32.6875	−117.1875	1.5	0.7	1.4	−2.7	−10.4	0.6	1.0	−1.2
3	New York, NY	40.6875	−74.0625	7.2	0.6	−0.5	60.0	14.0	0.7	−0.8	78.8
4	Miami, FL	25.6875	−80.3125	9.5	−0.6	0.1	−4.0	7.2	−1.3	−0.5	−5.3
5	Denver, CO	39.6875	−105.0625	−3.2	−0.5	0.2	−5.6	−10.2	−1.0	0.3	−9.2
