# Corrigendum: Predictability of Precipitation Over the Conterminous U.S. Based on the CMIP5 Multi-Model Ensemble

**DOI:** 10.1038/srep33618

**Published:** 2016-09-16

**Authors:** Mingkai Jiang, Benjamin S. Felzer, Dork Sahagian

Scientific Reports 6: Article number: 29962; 10.1038/srep29962 published online: 07 18 2016; updated: 09 16 2016.

This Article contains typographical errors in Reference 21.

“Ehasani, N., B. M. Fekete, C. J. Vorosmarty & Z. D. Tessler *A neural network based general reservoir operation scheme. Stochastic Environmental Research and Risk Assessment* **1**:16, doi: 10.1007/s00477-015-1147-9 (2015).”

should read:

“Ehsani, N., Fekete, B. M., Vörösmarty, C. J. & Tessler, Z. D. A neural network based general reservoir operation scheme. *Stochastic Environmental Research and Risk Assessment* **30**, 1151–1166, DOI: 10.1007/s00477-015-1147-9 (2016).”

