# Supplementary material for: Predictability of Precipitation Over the Conterminous U.S. Based on the CMIP5 Multi-Model Ensemble
Source: Sci Rep. 2016 Jul 18;6:29962. doi: 10.1038/srep29962 (PMC4947969; doi:10.1038/srep29962)
Supplement: Supplementary Information [file srep29962-s1.pdf]

PREDICTABILITY OF PRECIPITATION OVER THE CONTERMINOUS U.S. BASED ON THE CMIP5 MULTI-MODEL ENSEMBLE

Mingkai Jiang*[1], Benjamin S. Felzer[1], & Dork Sahagian[1]

1. Earth and Environmental Sciences, Lehigh University, 1 W Packer Avenue, Bethlehem, PA, United States, 18015.

* Corresponding author (mij212@lehigh.edu)

Supplementary Information

Description:

SI Text 1. Mathematical derivation of the Colwell index

Table S1. CMIP5 models and scenarios used in this study

Figure S1. Colwell index of predictability, constancy and seasonality, derived based on individual CMIP5 Spatially Downscaled but not Bias Corrected models over the historic period 1950 – 2005.

Figure S2. Taylor plot for the computed predictability, constancy, and seasonality scores across the US landscape for the historic period 1950-2005, based on Maurer monthly precipitation means (open black circle), CMIP5 Spatial Downscaled Bias Corrected (SDBC) multi-model ensemble means (closed black circle), and the individual CMIP5 SDBC model results (open red circles)

Figure S3. Gridded Z-scores for the percent difference between future under RCP4.5 scenario and historic (left panel), and between future under RCP 8.5 and historic (right panel), for the variables of predictability, constancy, seasonality, and mean annual precipitation over the conterminous US, based on ArcGIS computed Getis-Ord Gi* statistics.

Figure S4. Gridded P-scores for the percent difference between future under RCP4.5 scenario and historic (left panel), and between future under RCP 8.5 and historic (right panel), for the variables of predictability, constancy, seasonality, and mean annual precipitation over the conterminous US, based on ArcGIS computed Getis-Ord Gi* statistics.

SI Text 2. R codes used in this study

SI Text 1. Mathematical derivation of the Colwell index

Mathematically, the Colwell index is constructed based on a frequency matrix with *t* columns (months within a year) and *s* rows (categorical bins of precipitation magnitude). Let N_{ij} represent the number of years for which precipitation was at magnitude *i* in month *j*, column totals (X_j) are calculated as $X_j = \sum_{i=1}^{s} N_{ij}$, row totals as $Y_i = \sum_{j=1}^{t} N_{ij}$, and the grand total as $Z = \sum_i \sum_j N_{ij} = \sum_j X_j = \sum_i Y_i$. Uncertainty with respect to time is calculated as $H(X) = -\sum_{j=1}^{t} \frac{X_j}{Z} log \frac{X_j}{Z}$, uncertainty with respect to precipitation magnitude as $H(Y) = -\sum_{i=1}^{s} \frac{Y_i}{Z} log \frac{Y_i}{Z}$, and uncertainty with respect to the interaction of time and magnitude as $H(XY) = -\sum_i \sum_j \frac{N_{ij}}{Z} log \frac{N_{ij}}{Z}$. The conditional uncertainty with regard to magnitude, with time given, is defined as $H_X(Y) = H(XY) - H(X)$. When predictability is at its minimum, all magnitudes of precipitation are independent of time. In this case $H(X) = log\ t$, and $H(XY) = log\ st$, so that $H_X(Y) = log\ s$. The measure of predictability is therefore calculated as

$$P = 1 - \frac{H_X(Y)}{\log s} = 1 - \frac{H(XY) - H(X)}{\log s}$$

Constancy is minimized when row sums are all equal and maximized when all row totals but one are zero. Since *H(Y)* varies in precisely the opposite way, and its maximum value is log *s*, constancy is computed as $C = 1 - \frac{H(Y)}{\log s}$. Moreover, seasonality represents the dependence relationship between time and precipitation magnitude. The adjusted measure of seasonality based on mutual information (Jelineck, 1968) is calculated as $S =$

$\frac{H(X)+H(Y)-H(XY)}{\log s}$. Seasonality is minimized when all columns are homogenous. The details of the mathematical derivation are provided in Colwell (1974).

Reference

Colwell, R.K., 1974. Predictability, constancy, and contingency of periodic phenomena. Ecology, 55:1148-1153.

Jelinek, F., 1968. Probabilistic information theory: discrete and memoryless models. McGraw-Hill, New York, 609 pp.

Table S1. CMIP5 models and scenarios used in this study

Model Name	Institution	Scenario	Run
access1-0	Commonwealth Scientific and Industrial Research Organization (CSIRO) and Bureau of Meteorology (BOM), Australia	historic bcsd, sdnobc, rcp45, rcp85	r1i1p1
access1-3	same as above	historic bcsd, sdnobc, rcp45, rcp85	r1i1p1
bcc-csm1-1	Beijing Climate Center, China Meteorological Administration	historic bcsd, sdnobc, rcp45, rcp85	r1i1p1
bcc-csm1-1-m	same as above	historic bcsd, sdnobc, rcp45, rcp85	r1i1p1
canesm2	Canadian Centre for Climate Modelling and Analysis	historic bcsd, sdnobc, rcp45, rcp85	r1i1p1
ccsm4	University of Miami - RSMAS; National Center for Atmospheric Research	historic bcsd, sdnobc, rcp45, rcp85	r1i1p1
cesm1-bgc	Community Earth System Model Contributors	historic bcsd, sdnobc, rcp45, rcp85	r1i1p1
cesm1-cam5	same as above	historic bcsd, sdnobc, rcp45, rcp85	r1i1p1
cmcc-cm	Centro Euro-Mediterraneo per I Cambiamenti Climatici	historic bcsd, sdnobc, rcp45, rcp85	r1i1p1
cnrm-cm5	Centre National de Recherches Météorologiques / Centre Européen de Recherche et Formation Avancée en Calcul Scientifique	historic bcsd, sdnobc, rcp45, rcp85	r1i1p1
csiro-mk3-6-0	Commonwealth Scientific and Industrial Research Organization in collaboration with Queensland Climate Change Centre of Excellence	historic bcsd, sdnobc, rcp45, rcp85	r1i1p1
ec-earth	EC-EARTH consortium	historic bcsd, sdnobc, rcp45, rcp85	r12i1p1
fgoals-g2	LASG, Institute of Atmospheric Physics, Chinese Academy of Sciences and CESS,Tsinghua University	historic bcsd, sdnobc, rcp45, rcp85	r1i1p1
fgoals-s2	LASG, Institute of Atmospheric Physics, Chinese Academy of Sciences	historic bcsd, sdnobc, rcp45, rcp85	r2i1p1
fio-esm	The First Institute of Oceanography, SOA, China	historic sdnobc, rcp45, rcp85	r1i1p1
gfdl-cm3	NOAA Geophysical Fluid Dynamics Laboratory	historic bcsd, sdnobc, rcp45, rcp85	r1i1p1
gfdl-esm2g	same as above	historic bcsd, sdnobc, rcp45, rcp85	r1i1p1
gfdl-esm2m	same as above	historic bcsd, sdnobc, rcp45, rcp85	r1i1p1
giss-e2-r	NASA Goddard Institute for Space Studies	historic bcsd, sdnobc, rcp45, rcp85	r1i1p1
giss-e2-r-cc	same as above	historic bcsd, sdnobc, rcp45, rcp85	r1i1p1
hadcm3	Met Office Hadley Centre (additional HadGEM2-ES realizations contributed by Instituto Nacional de Pesquisas Espaciais)	historic bcsd, sdnobc, rcp45, rcp85	r1i1p1
hadgem2-cc	same as above	historic sdnobc, rcp45, rcp85	r1i1p1
hadgem2-ao	National Institute of Meteorological Research/Korea Meteorological Administration	historic bcsd, sdnobc, rcp45, rcp85	r1i1p1
inmcm4	Institute for Numerical Mathematics	historic bcsd, sdnobc, rcp45, rcp85	r1i1p1
ipsl-cm5a-lr	Institut Pierre-Simon Laplace	historic bcsd, sdnobc, rcp45, rcp85	r1i1p1
ipsl-cm5a-mr	same as above	historic bcsd, sdnobc, rcp45, rcp85	r1i1p1
ipsl-cm5b-lr	same as above	historic bcsd, sdnobc, rcp45, rcp85	r1i1p1

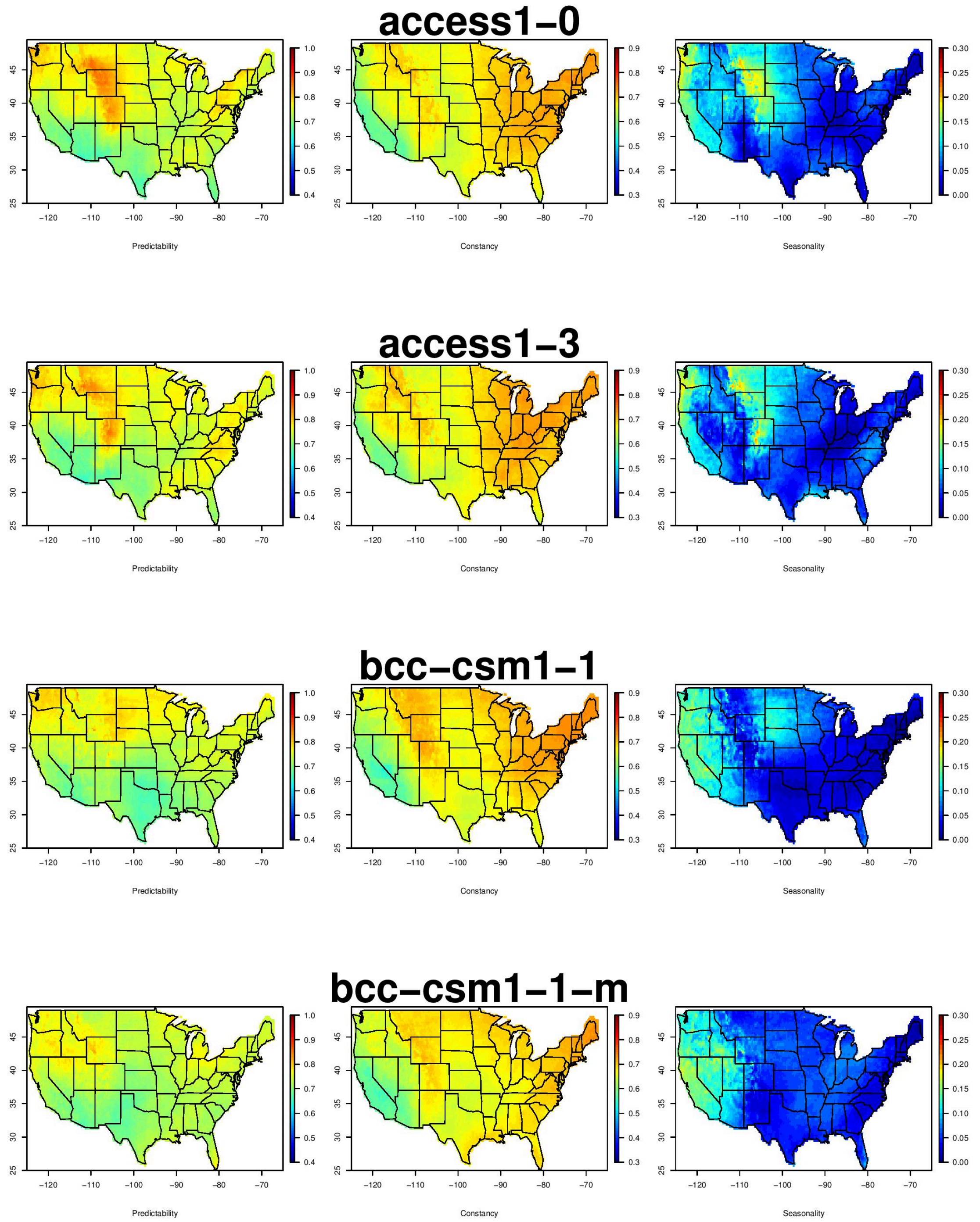
access1−0
access1−3
bcc−csm1−1
bcc−csm1−1−m
Predictability
Constancy
Seasonality

canesm2

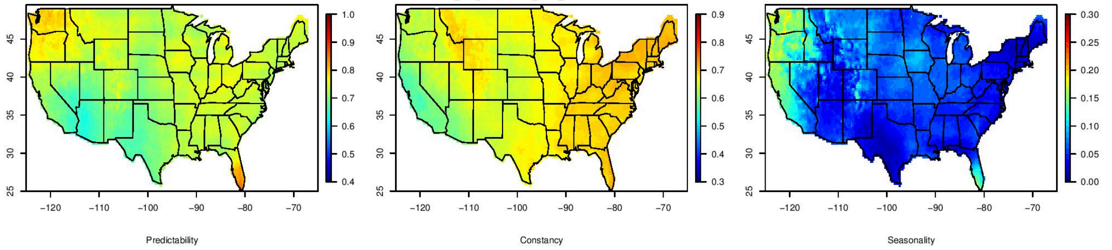

ccsm4

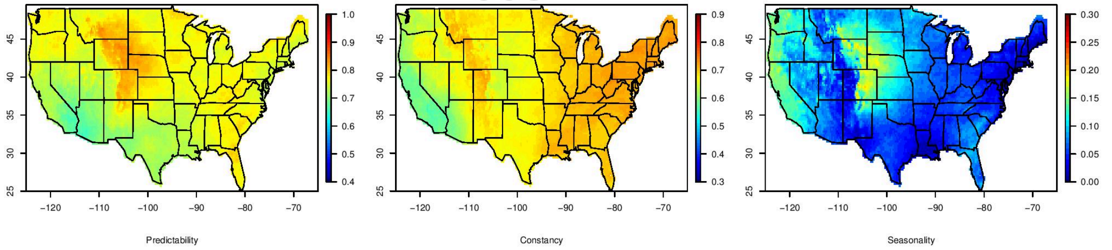

cesm1−bgc

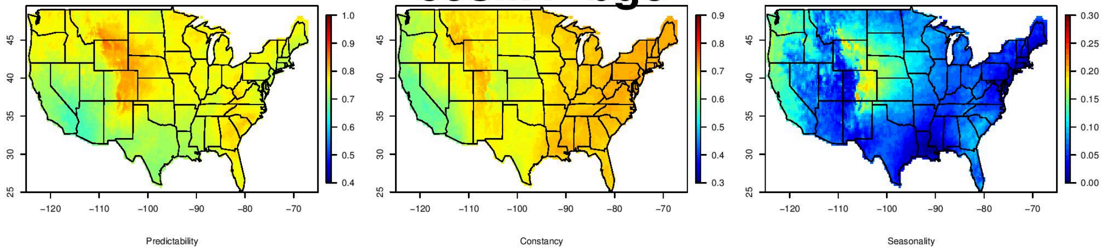

cesm1−cam5

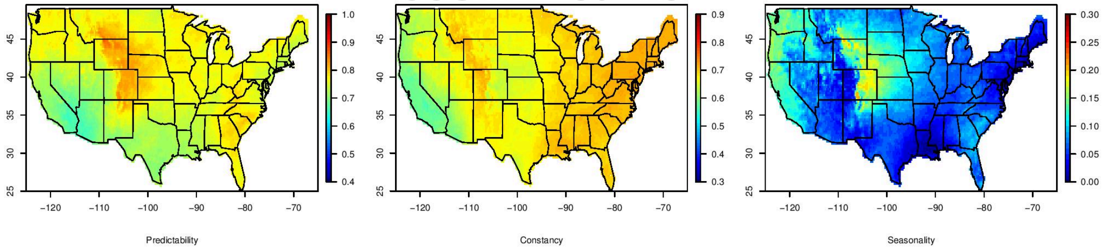

cmcc–cm

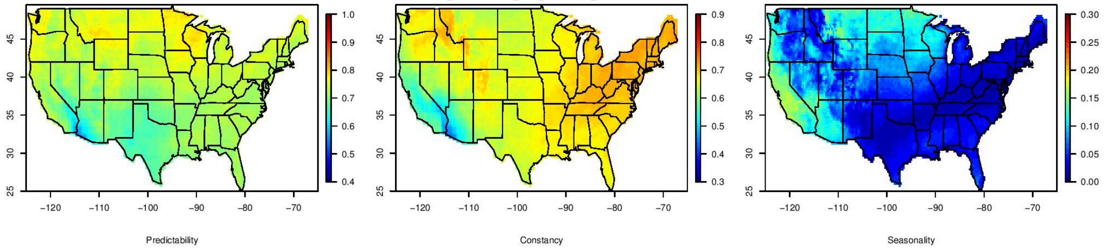

cnrm–cm5

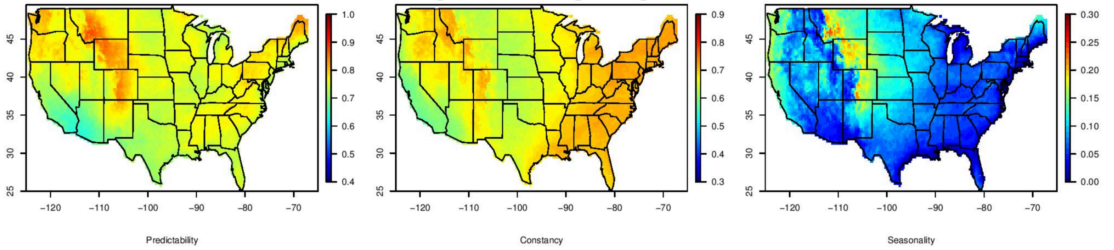

csiro–mk3–6–0

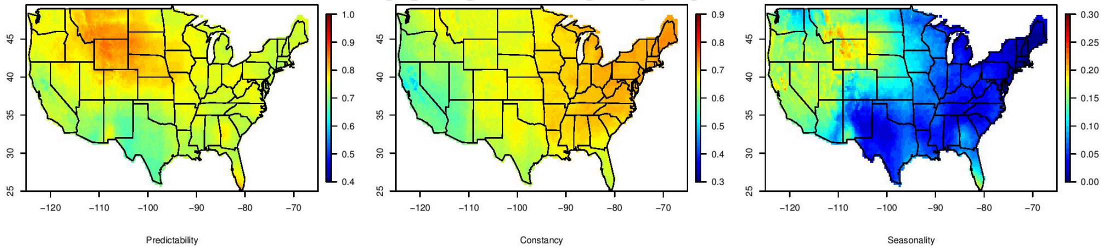

ec–earth

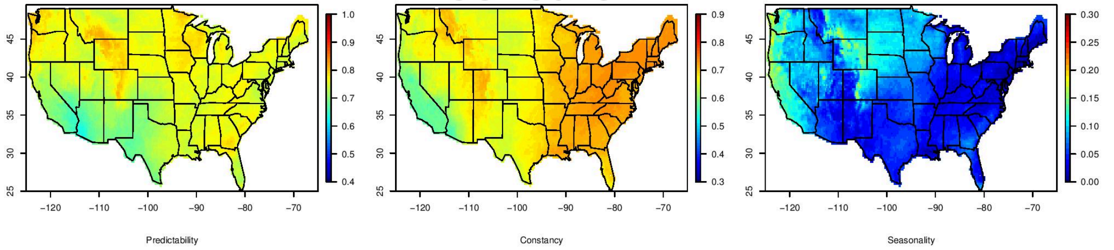

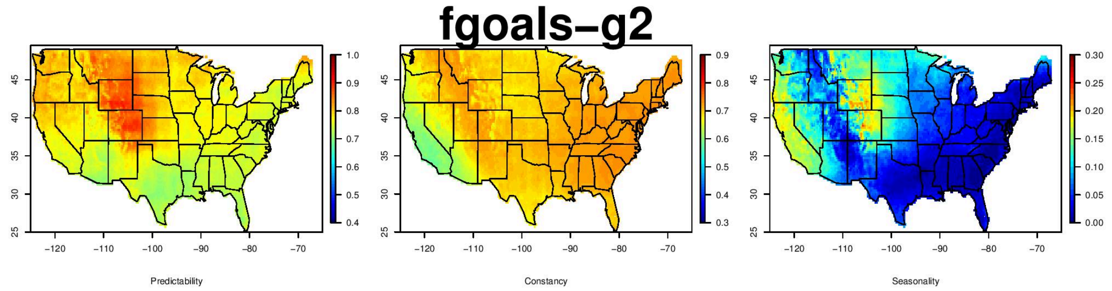
fgoals–g2
Predictability
Constancy
Seasonality

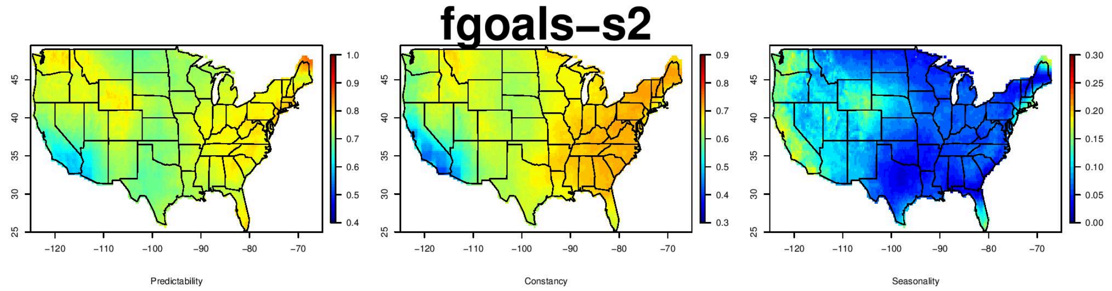
fgoals–s2
Predictability
Constancy
Seasonality

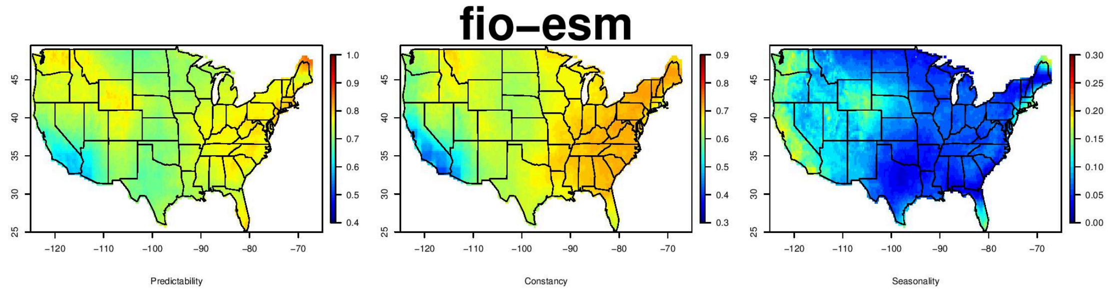
fio–esm
Predictability
Constancy
Seasonality

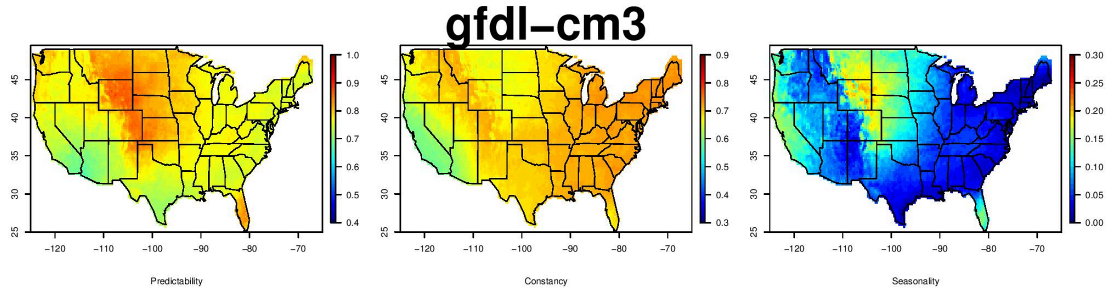
gfdl–cm3
Predictability
Constancy
Seasonality

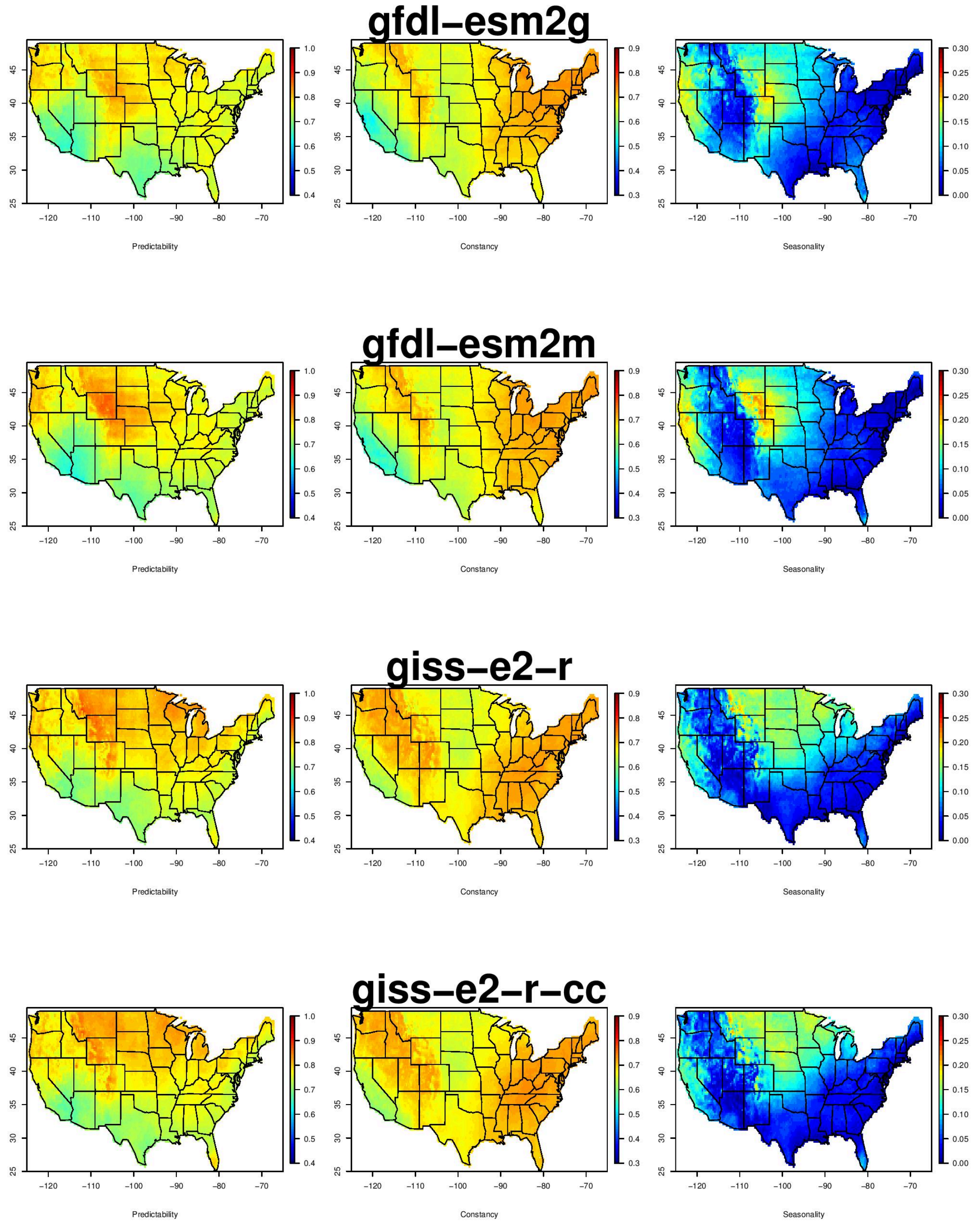
gfdl−esm2g
Predictability
Constancy
Seasonality
gfdl−esm2m
Predictability
Constancy
Seasonality
giss−e2−r
Predictability
Constancy
Seasonality
giss−e2−r−cc
Predictability
Constancy
Seasonality

hadcm3

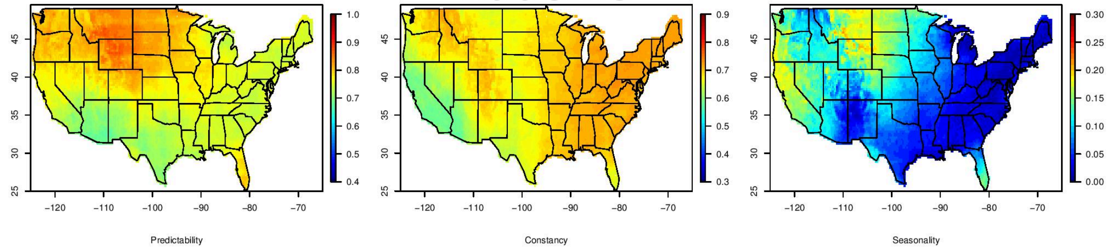

hadgem2−ao

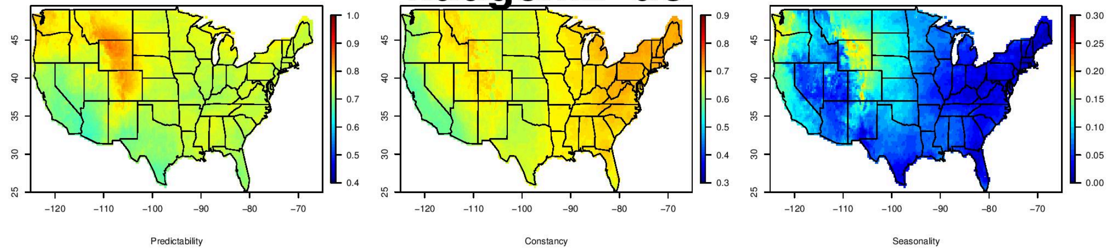

inmcm4

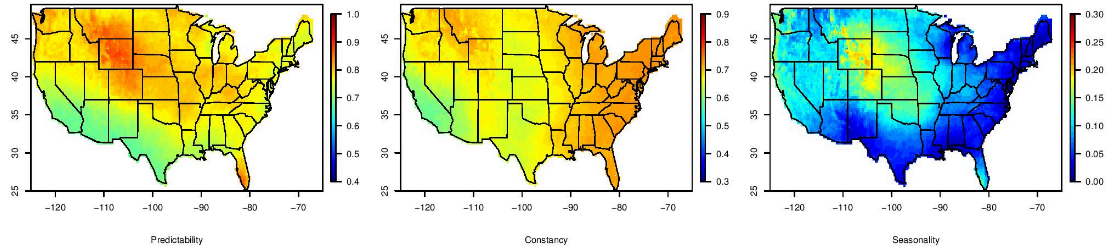

ipsl−cm5a−lr

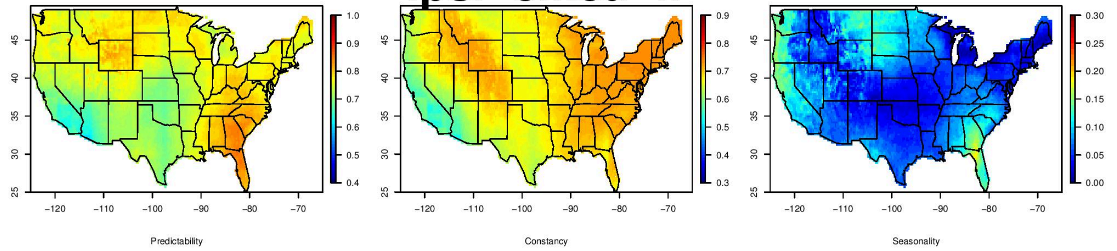

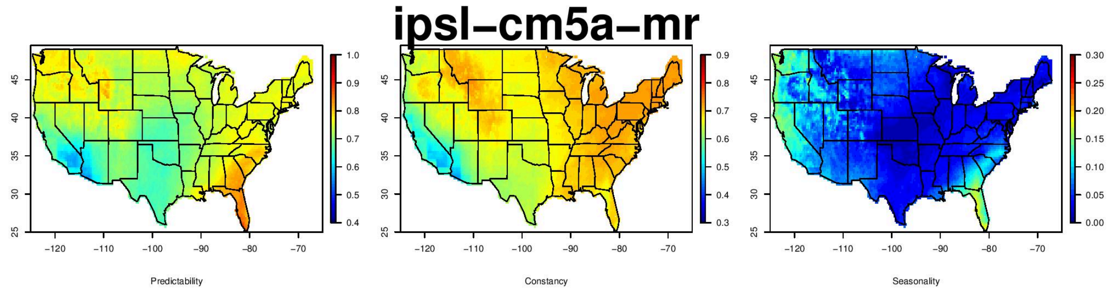
ipsl−cm5a−mr
Predictability
Constancy
Seasonality

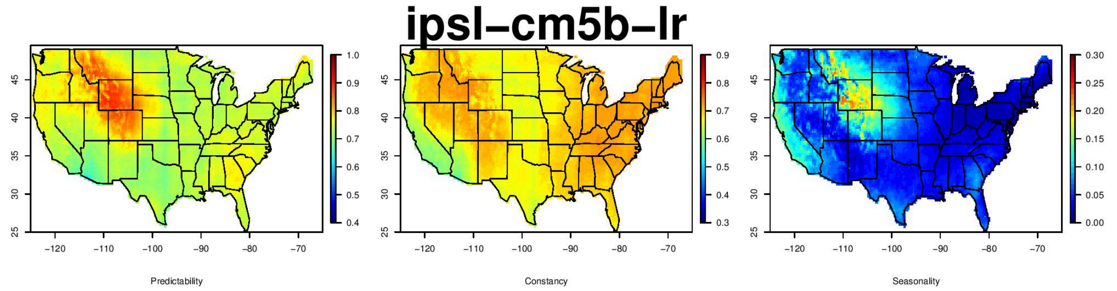
ipsl−cm5b−lr
Predictability
Constancy
Seasonality

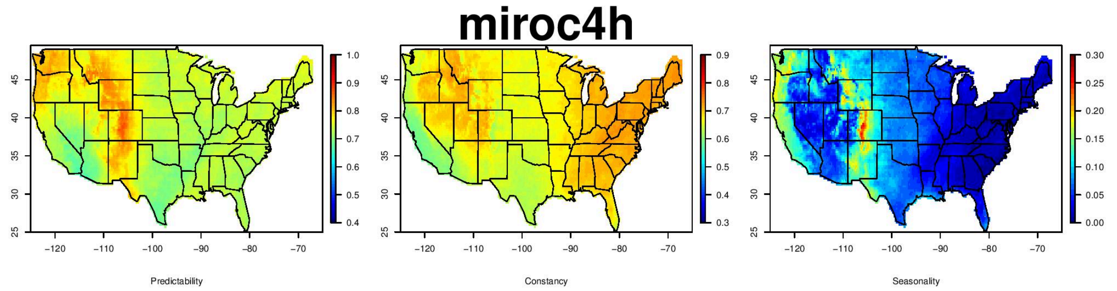
miroc4h
Predictability
Constancy
Seasonality

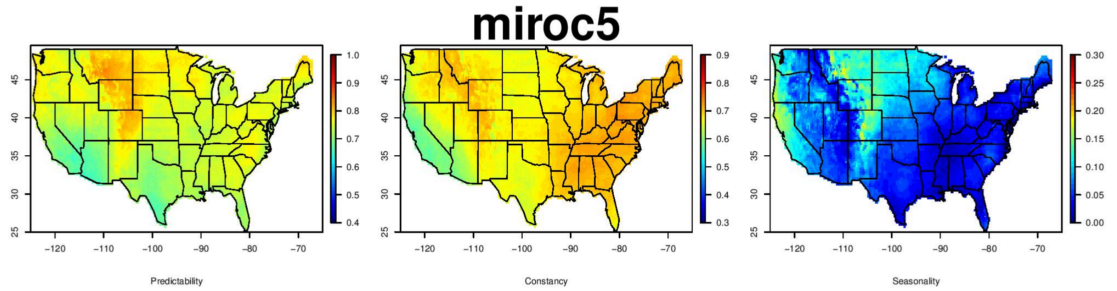
miroc5
Predictability
Constancy
Seasonality

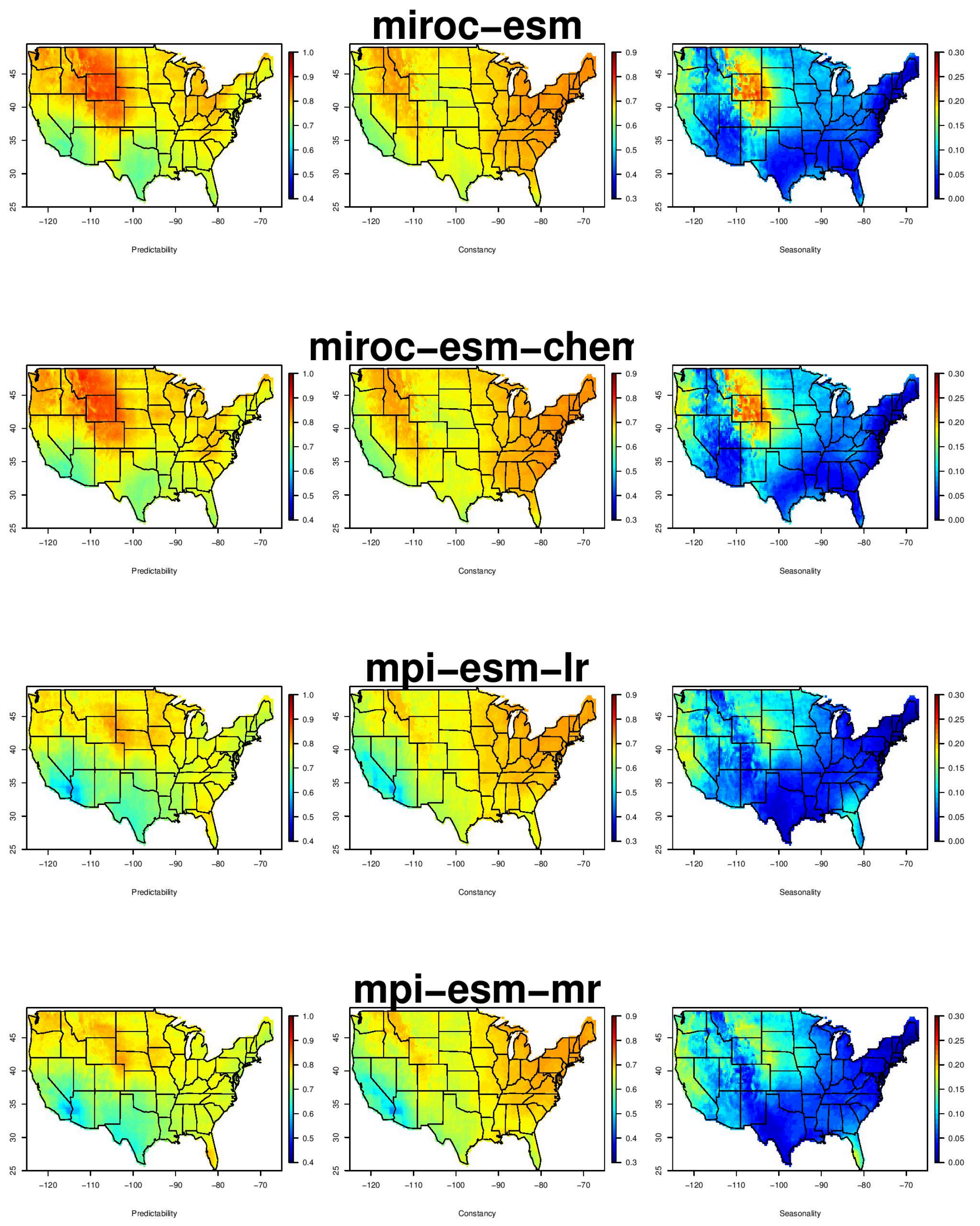
miroc–esm
Predictability
Constancy
Seasonality
miroc–esm–chem
Predictability
Constancy
Seasonality
mpi–esm–lr
Predictability
Constancy
Seasonality
mpi–esm–mr
Predictability
Constancy
Seasonality

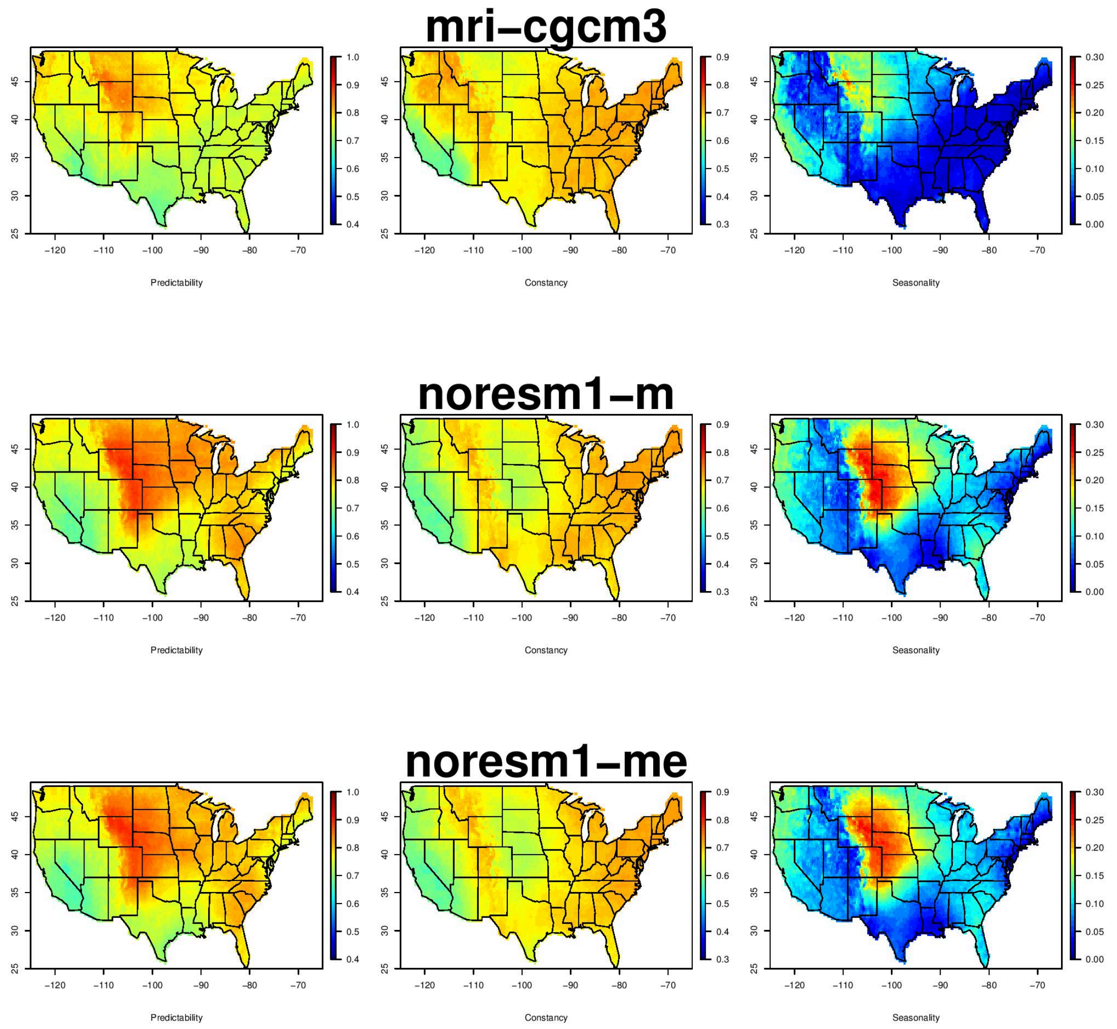

Figure S1. Colwell index of predictability, constancy and seasonality, derived based on individual CMIP5 Spatially Downscaled but not Bias Corrected models over the historic period 1950 – 2005. Figure was plotted in R Studio (V0.99, RStudio Inc. https://www.r-project.org).

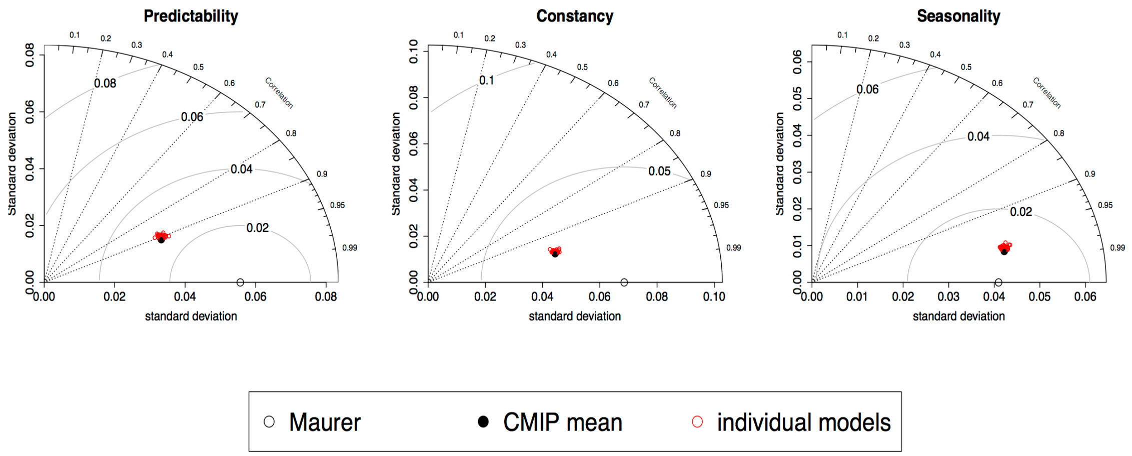

Figure S2. Taylor plot for the computed predictability, constancy, and seasonality scores across the US landscape for the historic period 1950-2005, based on Maurer monthly precipitation means (open black circle), CMIP5 Spatial Downscaled Bias Corrected (SDBC) multi-model ensemble means (closed black circle), and the individual CMIP5 SDBC model results (open red circles). Figure was plotted in R Studio (V0.99, RStudio Inc. https://www.r-project.org).

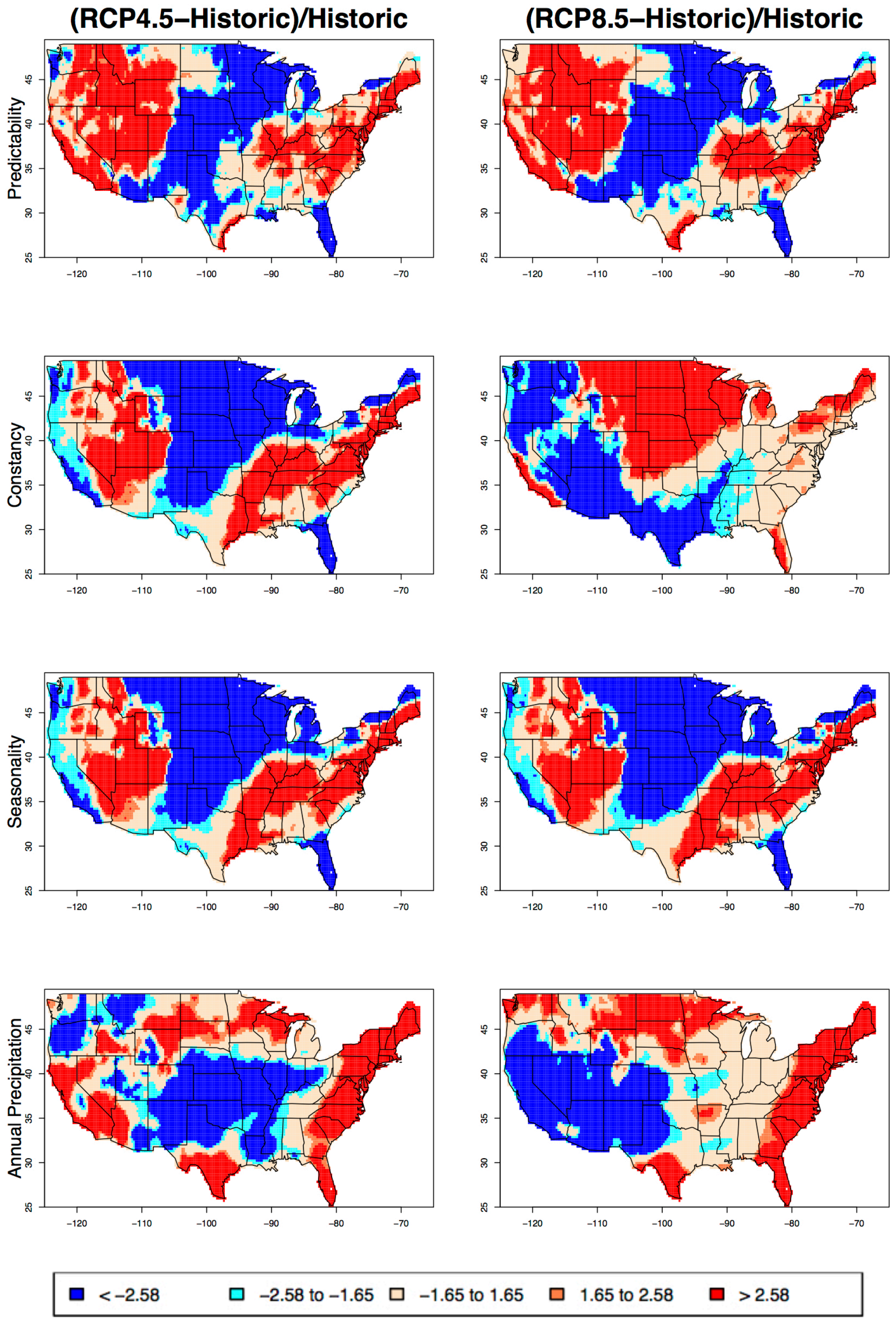

Figure S3. Gridded *Z*-scores for the percent difference between future under RCP4.5 scenario and historic (left panel), and between future under RCP 8.5 and historic (right panel), for the variables of predictability, constancy, seasonality, and mean annual

precipitation over the conterminous US, based on ArcGIS computed Getis-Ord Gi* statistics calculated from multi-model ensemble means. The Getis-Ord Gi* statistics provide a spatial analysis of hot spot clustering. The resultant Z-score informs where features with either high or low values cluster spatially. It is calculated by taking a standard deviation of the data (e.g. percent difference in predictability) for all grids across the US, and weighting according to hotspot/coldspot clustering. The standard deviation values provided in the figure are quantiles of a normal distribution. Figure was plotted in ArcGIS for Desktop (V10.0, ESRI 2011; http://www.esri.com/software/arcgis/arcgis-for-desktop). For more detailed description of the mathematics, please refer to: http://pro.arcgis.com/en/pro-app/tool-reference/spatial-statistics/what-is-a-z-score-what-is-a-p-value.htm

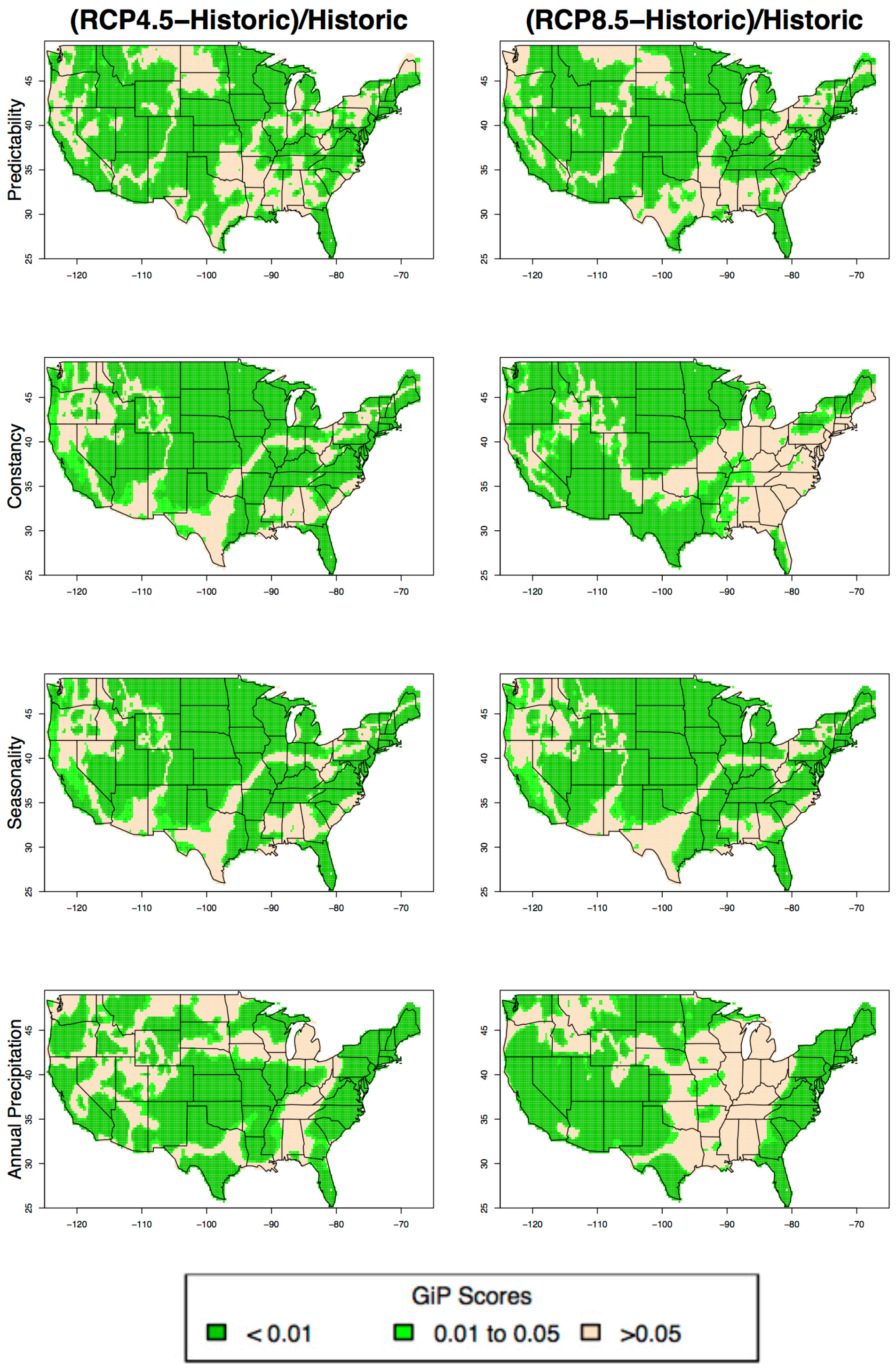

Figure S4. Gridded P-scores for the percent difference between future under the RCP4.5 scenario and historic (left panel), and between future under the RCP 8.5 and historic (right panel), for the variables of predictability, constancy, seasonality, and mean annual

precipitation over the conterminous US, based on ArcGIS computed Getis-Ord Gi* statistics calculated from multi-model ensemble means. The Getis-Ord Gi* statistics provide a spatial analysis of hot spot clustering. The p-values evaluate whether the observed spatial pattern is randomly created or not. Figure was plotted in ArcGIS for Desktop (V10.0, ESRI 2011; http://www.esri.com/software/arcgis/arcgis-for-desktop). For more detailed description, please refer to: http://pro.arcgis.com/en/pro-app/tool-reference/spatial-statistics/what-is-a-z-score-what-is-a-p-value.htm
